# Neuronavigation. Principles. Surgical technique.

**Published:** 2009

**Authors:** Marcel Ivanov, Alexandru Vlad Ciurea

**Affiliations:** * „Prof. Dr. N. Oblu” Clinical Emergency Hospital, Iasi; ** „Bagdasar-Arseni” Clinical Emergency Hospital, Bucharest

**Keywords:** Neuronavigation, Computer assisted surgery

## Abstract

Neuronavigation and stereotaxy are techniques designed to help neurosurgeons precisely localize different intracerebral pathological processes by using a set of preoperative images (CT, MRI, fMRI, PET, SPECT etc.). The development of computer assisted surgery was possible only after a significant technological progress, especially in the area of informatics and imagistics. The main indications of neuronavigation are represented by the targeting of small and deep intracerebral lesions and choosing the best way to treat them, in order to preserve the neurological function. Stereotaxis also allows lesioning or stimulation of basal ganglia for the treatment of movement disorders. These techniques can bring an important amount of confort both to the patient and to the neurosurgeon.

Neuronavigation was introduced in Romania around 2003, in four neurosurgical centers. We present our five-years experience in neuronavigation and describe the main principles and surgical techniques.

## Introduction

From the early beginning of modern neurosurgery (the end of the 19th century) the neurosurgical progress had tight connections with the intracranian localization possibilities. Knowledge of spatial relationships of the lesion inside the skull and the development of minimally invasive approaches have an essential contribution to the decrease of mortality and morbidity in neurosurgical procedures. The localization means the answer to two questions: 

1. Where are the lesions or the functional area situated inside the skull?

2. How can this be found during surgical procedure?

The answer to the first question was possible due to the development of modern neuroimagistic techniques (CT, MRI). The answer to the second question is more complex and had a longer evolution. 

At the beginning of the 20th century the diagnosis and localization of the lesion was possible almost exclusively as a result of the study of neurological symptoms without any possibility to refer to radiological images.

The first technique, described by Dandy in 1918 [**1**], was ventriculography – air injection and later, there was the contrast substance which was pumped in the ventricles so as to allow their visualization on the skull X-ray. Lesions from the vicinity of the ventricles could be localized depending on the form and displacement of the ventricles. Egas Moniz is said to have introduced angiography in 1927. This technique allowed the localization of intracerebral lesions both directly, by visualizing different vascular malformations and indirectly, by studying the vessel displacement from the normal anatomy in different cerebral lobes. Direct visualization of the brain was possible only after the introduction of the computer tomography (CT) in 1973, by Hounsfield [**6**]. 

Intraoperative localization technique, including craniotomy position was based on knowledge of specific bone landmarks from the skull, as coronar suture, external occipital protuberance, pterion, etc and on neurosurgeon’s ability and experience to perform a 3D orientation. After opening the skull other landmarks from its base were represented by other anatomical structures: nerves, vessels and specific bony landmarks. This “anatomic” localization method was and is still considered the “golden standard” both before and after CT/MRI era. Microneurosurgeons use this detailed anatomical information for a better identification and planning of some complex approaches [**3**,**11**,**12**]. 

Parallel to anatomical localization, yet from the beginning of neurosurgery there was a tendency to define in advance the anatomical and pathological structures, using some mechanical tools. The purpose of these tools was to establish a precise approach to the target providing objective information, irrespective of individual surgical aptitudes. A stereotactic frame was based on rigid coordinate system, where target and trajectory were calculated on the ground of imagistic information. However, neurosurgeons feel more comfortable when identifying the anatomical landmarks rather than the stereotactic frame. The reason is not only the idea that neurosurgeons like knowing each next step based on a continuously changing intraoperative situation, but also the fact that they believe more in what they can see than in what a computer might display. 

Robotic techniques or those based on stereotactic frame have pushed neurosurgeons to adopt neurosurgical techniques to rigid stereotactic frames which were functioning with extremely high accuracy, but with reduced flexibility. 

## Technique description

For all the cases operated on in the Department of Neurosurgery in Iasi, we used Radionics neuronavigation system. In order to learn the working principles of other neuronavigation systems (*BrainLab, Medtronic Stealth*), a study and training visits to BrainLab, Munchen, Germany (the leading corporation in the field of neuronavigation) and other neurosurgical centers from USA and Europe have been made.

Each neuronavigation system follows the same steps in order to link surgical procedure to the images obtained both pre- and intraoperatevely:

*- obtaining preoperative images*;

*- registration*;

*- intraoperative localization*;

*- intraoperative control*;

*- obtaining intraoperative images and fusion with preoperative ones*;

*- visualization and surgery*.

## Obtaining preoperative images

Due to the fact that neuronavigation is a 3D process, optic images used by neuronavigation must be 3D. At the beginning, large use of neuronavigation was limited by the fact that imaging technology was limited by the 2D projection on conventional X-ray scan. After developing the 3D imaging (helical CT, MRI), stereotactic techniques became much more popular. 

3D CT image-set represents a number of images taken at a precise interval of time and a specific distance between them. The smaller the distance between images, the better the accuracy of the obtained 3D image. These images can fuse in order to obtain a virtual threedimensional object. The technological progress not only allowed the visualization of anatomical structures, but also some specific cerebral functions. In order to integrate and fuse these information together, giving the neurosurgeon the possibility to operate both on the base of anatomical and functional abnormalities [**7**]. The imaging that gives us functional information is represented by PET, SPECT, fMRI and magnetoencephalography. The main benefit of this possibility to combine functional and anatomical images is represented by the possibility to keep eloquent brain function located in the vicinity of the lesion. On the other hand, apparently normal (from the anatomical point of view) brain tissue may be functionally abnormal and generate epilepsy. Resection of such spots with preservation of normal function guided by neuronavigation is another new technique used in neurosurgery [**14**].

**Fig. 1:**
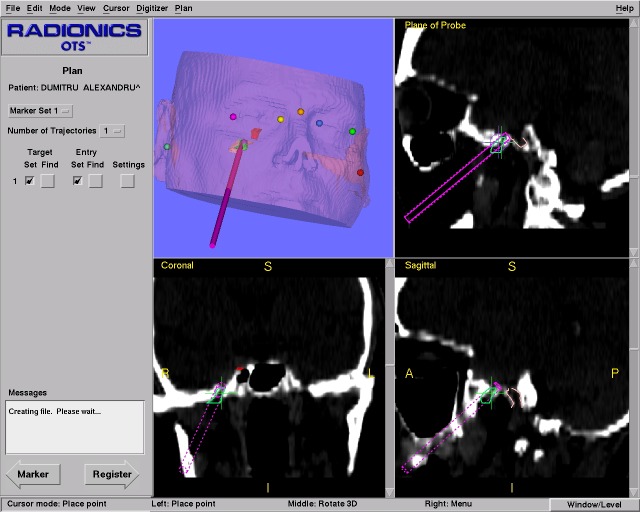
Patient’s registration using anatomical pair-points.

 A difficulty that can appear when using functional imaging is represented by low resolution of functional images. For instance, PET, which has the highest resolution among imaging techniques, has a resolution of only 256 × 256 pixels and a thickness of 3.7 mm compared to CT images which usually have a resolution of 512×512 pixels and the thickness of 1 mm between scans. To analyze these images and use them intraoperatively with a good accuracy, they must be adapted to information set with a higher anatomical resolution. This procedure is called *image fusion*. It can be performed by a neurosurgeon manually by superposing the correspondent images in both sets of images (for example, PET and MRI/CT). The main points to superpose are: eyeball, cornea, white comissure, skin markers etc. This registration of pair-points can also be performed automatically. Another possibility is to superpose the visible contours in both data sets.

Technological progress facilitated the development of CT-angio and MR-angio and permited the introduction of vascular information in the navigation system [**13**]. 

The images can be transferred in the navigation system via computer network or by using a mobile support, such as a CD or a memory stick (in BrainLab). These images should be in DICOM format.

## Patient’s registration

One of the very important steps in operating with neuronavigation is patient’s registration. An accurate patient’s registration will precisely facilitate the operation and recognition of the instruments in the operating field, by the neuronavigation system. 

The registration technique is similar to the fusion technique described above and, in most of our cases was performed by using fiducials – skin markers attached to the patient’s head before obtaining CT or MRI images. In order to increase the accuracy it is important to use as many fiducials as possible (minimum 6, optimal 12-15). The placement of the fiducials on different levels and not in one line is another factor that contributes to improved accuracy of the procedure. It is important to inform the patient to keep the fiducials attached to the skin until he gets into the operating room and also not to remove them at the end of CT/MRI scan. Sometimes a helmet to protect them may be useful. As a safety measure, the center of the fiducial may be pointed out with a marker in case it is detached.

Another possibility that we used in our department is the anatomical pair-point registration. On the images, external anatomical landmarks, like tip of the nose, naso-labial angle, superior and inferior limit of the ear, internal and external limit of the eyes, will be selected. The use of a higher number of landmarks is recommended.

The third method of registration used in our department is recognition of the face contour. A large number of points (more than 100) are hierarchically selected around the nose, forehead, ear, scalp, periorbital, etc. The sum of these points will give a face countour recognized by the navigation software and superposed over 3D virtual face reconstrcuction. The accuracy of this method is considered to be low, but it increases the more the number of registered points raises. Besides this method of touching the face contour with an instrument recognized by navigation, BrainLab gives another method that uses a similar principle but, instead of an instrument touching the face, a laser is used and the light reflection is recognized by a special camera (OTS – Optic Tracking Device) (**Fig. 3**).

**Fig. 2:**
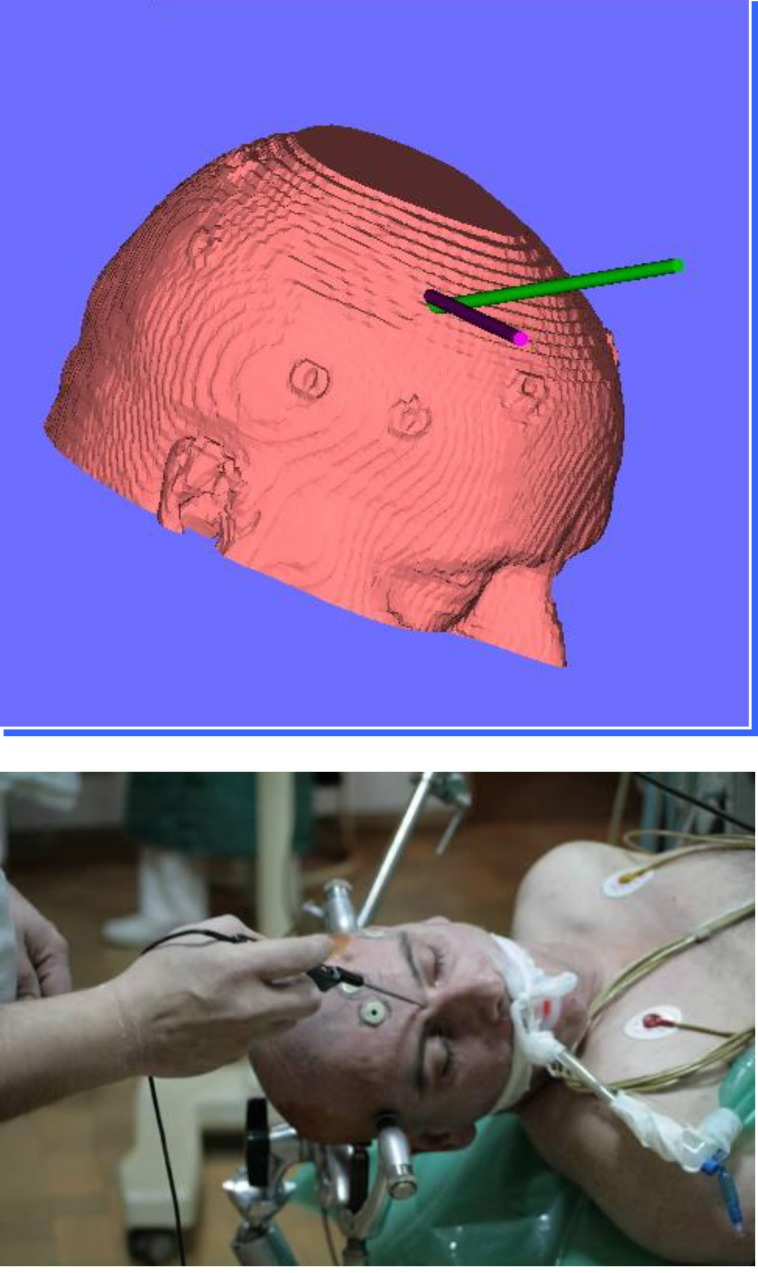
Pacient’s registration using skin markers (fiducials)

Due to relative mobility of the skin (and the attached markers) to the bone, the final accuracy given by navigation is about 3-7 mm. This value can be improved by the increase of fiducials used or by another reference marker implantation (screwing) into the skull.

Patient’s registration is performed in the operating theatre, on the operating table, after his head is fixed in a Mayfield frame which has an arch with LEDs attached. It is extremely important to keep the position of these three elements (head, frame, and arch with LEDs) relative, stable and constant. The modification of the position of any of these elements can lead to serious errors and failure of the navigation. After the registration, but before starting the operation, it is advisable to perform a second check by touching different parts of the patient’s face with an instrument recognized by navigation. The operation of aquiring the virtual patient’s face must be reproduced on the navigation monitor. The nose and external auditore meatus are the most common anatomical landmarks used for this purpose [**5**,**8**,**10**,**15**].

**Fig. 3:**
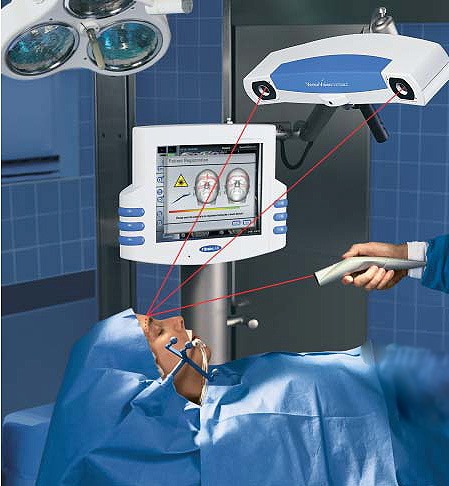
Laser face contour recognition and registration.

## Localization

There are several ways to recognize the intraoperative instrument by navigation. This function is performed by a 3D-digitizer, which follows the emitted or reflected signal that comes from an instrument. 

First navigation prototypes used microphones as detectors and *ultrasound* generating electrodes as emitters. This type of digitizer needs a free unobstructed field between emitter and receiver. The use of sound for localization was suboptimal due to generated echoes. Moreover, the propagation of the speed of sound depends on ambiance temperature. 

Optic localization techniques are similar to those used in ultrasound equipment, with a difference that some of the problems caused by sound properties were sorted out. This tool is based on spatial triangulation using cylindrical lenses to focalize the light emitted by LEDs. Each element will determine the position of one LED in a single plan. Comparing the information received from those three cameras, a spatial position, with a mechanical accuracy of 0.4 mm, is possible. Although keeping an unobstructed field remains a criteria that needs to be respected, the problems generated by the operating theatre temperature and echoes were eliminated.

The reference frame for cranial application has an arch form with more LEDs on its surface. This reference frame is directly attached to the Mayfield frame and it stays in this position during the entire surgical procedure. The reference frame has to be attached to the Mayfield frame before the patient’s registration. 

A third component of this system (along with reference frame and optic digitizer camera) is the surgical instrument to which a LED’s equipped accessory tool is attached. These LEDs are placed at specific and fixed distance between each other and emit or reflect light during the surgical procedure. They must always be directed to the camera (OTS) to be recognized by the navigation system.

**Fig. 4:**
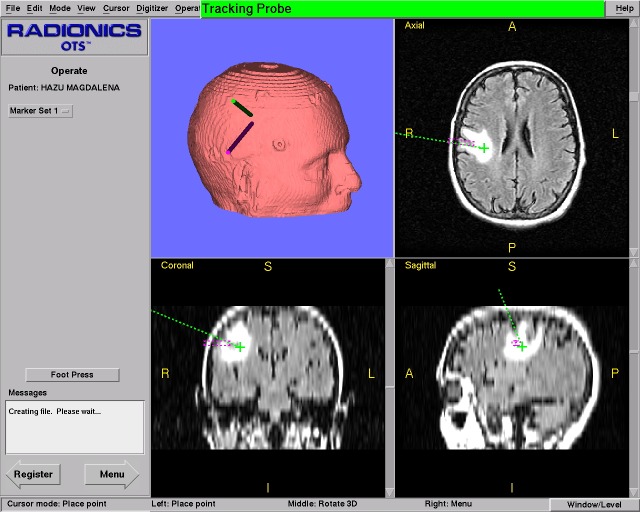
3D, axial, coronal and sagittal intraoperative planes seen on the monitor.

All the imagistic manipulations and surgical localizations are performed by the UNIX based working station. By default, three images corresponding to axial, sagittal and coronar planes along with a 3D reconstruction are shown on the monitor. The three standard plans can be changed with other plans, such as the plane parallel to the instrument’s axis or the plane perpendicular to the instruments’ axis at the tip level (“probe’s eye”). All the images may be saved in JPEG format anytime during the surgery. Surgical instrument position is projected on the screen as a virtual instrument of green color (**Fig. 4**). The surgeon can select the target and the optimal approach preoperatively and follow it or be guided by these images during the surgery [**5**,**8**,**10**,**15**].

## Visualization

During the entire surgical procedure the exact position of the instrument’s tip can be visualized on the monitor in the three default plans previously discussed about. In order to facilitate the surgeon’s orientation, other plans can be created or the instrument’s orientation in a 3D view can be performed. Surgical target can be selected and a contour with a defined color can be performed around the target. If necessary, the skin or the skull on the monitor may become transparent or semitransparent (**Fig. 4**).

Sometimes it may be difficult to follow both the real situation in the skull and the image on the monitor. We prefer placing the monitor at the pacient’s legs. This allows it to be seen both by the surgeon and the assistants. 

Another option to combine images is represented by the projection of the virtual images into the microscope’s ocular, over the real image.

## Conclusions

Neuronavigation is a tool that provides numerous advantages to the neurosurgeon: a more accurate planning approach, the performing of a smaller approach, high precision intraoperative localization of different anatomical structures, whenever their preserving is desired. This technique, although apparently very complex in the beginning, becomes very user-friendly and positively appreciated by everyone who uses it gradually, after going through a relatively short-time learning curve. Its value is even higher in the case of young neurosurgeons, who are in a process of learning and accumulation of 3D neurosurgical experience.

Neuronavigation can be compared with a GPS equipment, which is so popular today among car-drivers. Usually, most of the people do not need this device as long as they are driving a car in a familiar city and they use the same streets everyday. But, if they enter an unknown territory (or very little known) the value of this tool is extremely high as it helps them reach the target by using the most convenient (shortest or safest) way.

In our opinion neuronavigation and stereotaxy use may be and must be extended to a larger number of neurosurgical procedures for brain tumors and for functional neurosurgery. This modern technology brings an important amount of comfort and safety both to the patient and neurosurgeon.
